# Cocaine- and opiate-related fatal overdose in New York City, 1990–2000

**DOI:** 10.1186/1471-2458-7-31

**Published:** 2007-03-09

**Authors:** Kyle T Bernstein, Angela Bucciarelli, Tinka Markham Piper, Charles Gross, Ken Tardiff, Sandro Galea

**Affiliations:** 1 Department of Emergency Medicine, School of Medicine, New York University. 462 First Ave, 3^rd ^Floor, Room A345, NY, NY 10003. USA; 2 Center for Urban Epidemiologic Studies, New York Academy of Medicine, 1216 Fifth Ave, NY, NY 10029, USA; 3 Department of Psychiatry, Weill Medical College, Cornell University, 525 E. 68th St, NY, NY 10021 USA; 4 Department of Epidemiology, School of Public Health, University of Michigan. 1214 South University, Ann Arbor, Michigan, 48104-2548, USA; 5 Department of Epidemiology, Columbia University Mailman School of Public Health. 722 168th St, New York, New York 10032, USA

## Abstract

**Background:**

In New York City (NYC), the annual mortality rate is higher for accidental drug overdoses than for homicides; cocaine and opiates are the drugs most frequently associated with drug overdose deaths. We assessed trends and correlates of cocaine- and opiate-related overdose deaths in NYC during 1990–2000.

**Methods:**

Data were collected from the NYC Office of the Chief Medical Examiner (OCME) on all fatal drug overdoses involving cocaine and/or opiates that occurred between 1990–2000 (n = 8,774) and classified into three mutually exclusive groups (cocaine only; opiates-only; cocaine and opiates). Risk factors for accidental overdose were examined in the three groups and compared using multinomial logistic regression.

**Results:**

Overall, among decedents ages 15–64, 2,392 (27.3%) were attributed to cocaine only and 2,825 (32.2%) were attributed to opiates-only. During the interval studied, the percentage of drug overdose deaths attributed to cocaine only fell from 29.2% to 23.6% while the percentage of overdose deaths attributed to opiates-only rose from 30.6% to 40.1%. Compared to New Yorkers who fatally overdosed from opiates-only, fatal overdose attributed to cocaine-only was associated with being male (OR = 0.71, 95% CI 0.62–0.82), Black (OR = 4.73, 95% CI 4.08–5.49) or Hispanic (OR = 1.51, 95% CI 1.29–1.76), an overdose outside of a residence or building (OR = 1.34, 95% CI 1.06–1.68), having alcohol detected at autopsy (OR = 0.50, 95% CI 0.44–0.56) and older age (55–64) (OR = 2.53 95% CI 1.70–3.75)).

**Conclusion:**

As interventions to prevent fatal overdose become more targeted and drug specific, understanding the different populations at risk for different drug-related overdoses will become more critical.

## Background

Illegal drug users experience mortality rates greater than those among the general population[1]. In the United States and Europe, mortality among drug users has been estimated to be at least nearly seven times that of the general population[2-5]. While the causes of mortality among drug using populations are varied and include trauma [6] and HIV/AIDS[7], overdose is an important contributor to mortality in this group [8-10]. In 2003, non-medically related drug toxicity was the tenth leading cause of mortality in New York City (NYC) [11].

The overdose experiences of heroin users have been extensively studied [12-16]. Furthermore, the development of effective interventions to reduce accidental opiate overdose death, such as the provision of naloxone and the creation of safe injecting spaces have also been described [17-19]. Several review articles have been published synthesizing what we know about opiate overdose and its related mortality [20-22]. However, a number of studies have shown that multi-drug use contributes dramatically to overdose death [15,23,24], and in several cities, cocaine is suspected to be a considerable contributor to accidental overdose mortality[9].

While cocaine overdose is prevalent, there is a paucity of research that has examined the relative contribution of cocaine to overdose mortality. One of the few studies that examined fatal cocaine overdoses found that the route of administration for over 85% of decedents was injection, over half of the deaths occurred during a weekend and 41% of deaths occurred in the presence of others [25]. Therefore, although cocaine is probably an important contributor to overdose deaths in many urban areas[26], there is little research that has systematically explored the characteristics of decedents whose death is attributed to cocaine overdose[9].

In NYC, previous studies have documented the importance of polydrug overdose to overall overdoses [9] and racial/ethnic disparities in overdose fatalities [10]. However, there have been no papers of which we are aware that have studied the factors associated with cocaine vs. opiate-related overdoses in NYC or elsewhere. While the importance of polydrug overdose cannot be understated, as interventions for the prevention of fatal overdose become more targeted, such as naloxone for opiate overdoses [17,19,22,27-29], a clearer understanding of the importance of opiate and cocaine fatal overdose as distinct events increase. In this study, we examined all accidental overdose deaths in NYC between 1990 and 2000 to characterize differences in cocaine- and opiate-related drug overdoses. In NYC, mortality related to drug overdose is of substantial public health concern [30]. Therefore, the purpose of this study was to explore the epidemiologic correlates of cocaine- and opiate only related overdose. Furthermore, we sought to examine whether these correlates were different between those decedents who fatally overdosed with both cocaine and opiates, cocaine alone, and opiates alone. Understanding these epidemiologic differences may prove critical in the development of successful, innovative interventions and prevention messages that can reduce overdose-related mortality.

## Methods

### Study Design

All cases of fatal accidental drug overdose occurring in adults aged 15–64 years in NYC between 1990 and 2000 were identified through manual review of medical files at the Office of the Chief Medical Examiner of NYC (OCME).

### Study Population and Protocol

The OCME is responsible for assessing all deaths of persons believed to have died in an unnatural manner in NYC. Therefore, all overdose deaths in NYC would have been reviewed by the OCME and included in this chart abstraction.

The OCME investigators use the decedent's medical history, the circumstances and environment of the fatality, autopsy findings, and laboratory data in attributing cause of death and other criteria to each case reviewed. Deaths determined to be due to either suicide or homicide are not considered accidents by the OCME and hence are not included in this analysis. Data regarding age, gender, race/ethnicity, and residence were collected for all decedents from OCME files.

Rate of autopsy vary by manner of death. Between 1990 and 2000 approximately 80% of accidents were autopsied. All autopsied cases undergo toxicological screenings. Blood and urine samples were obtained at autopsy and stored at 4°C until they were assayed. Opiates refer to entire class of opiates (methadone, morphine, etc) and not just heroin. Further details on these data collection and toxicological methods have been presented elsewhere[31].

### Data Analysis

Analyses were conducted for all accidental overdose deaths involving cocaine and/or opiates. Between 1990 and 2000, 375 overdose deaths were identified that involved neither cocaine nor opiates; these were excluded from the analysis. We first described characteristics of all fatal overdoses in NYC between 1990 and 2000, including age, race/ethnicity, and gender of the decedents as well as drug and alcohol toxicities. We limited these analyses to all accidental drug overdose decedents aged 15–64 years old. Younger (<15) and older (65+) decedents were excluded from this analysis because of the potentially different drug use patterns and practices in these groups. In order to assess the epidemiology of opiate-related and cocaine-related overdose deaths we categorized decedents into three mutually exclusive categories: (a) overdose deaths in which cocaine (not in the presence of opiates) was the cause of death; these are referred to as "cocaine only" overdose deaths in the rest of the paper although we note that other drugs (except opiates) may also have been contributors of cause of death; and (b) overdose deaths in which opiates (without the presence of cocaine) were the cause of death; these are referred to as "opiate only" overdose deaths; (c) and overdose deaths in which both cocaine and opiates were the cause of death; these are referred to as "cocaine and opiate" overdose deaths. Deaths in which neither cocaine nor opiates were the cause of death were eliminated from the analysis. Decedents were categorized with respect only to cocaine and opiate presence at autopsy; other drug involvement was noted but did not impact on the above categorizations. For example, a decedent who had cocaine, opiates, and methamphetamines noted at autopsy, would be classified as cocaine and opiate overdose death; a decedent with cocaine and methamphetamines found at autopsy would be considered a cocaine only overdose death. One objective of this analysis was to determine if the epidemiologic characteristics of these three groups differed.

We calculated the total number and proportion of overdose deaths attributed to cocaine and opiates in NYC from 1990 through 2000. χ^2 ^statistics were used to examine trends in the number and proportion of cocaine excluding opiate and heroin-only induced overdose deaths. We described relevant characteristics of all accidental drug overdose deaths. Characteristics of interest included sex, race/ethnicity, age, borough of death, year of death, the toxicological presence of cannabis or alcohol, and the time and location of death. χ^2 ^statistics were used to assess bivariate associations between decedent characteristics of interest and the likelihood of cocaine only, opiate only, and cocaine and opiate induced overdose deaths.

The objective of this analysis was to examine differences between the three groups of decedents (cocaine only, opiate only, and cocaine and opiate). This was explored through two types of multivariate modeling. First, all covariates that were statistically significant at the p ≤ 0.05 level were included in multivariate logistic regression models. Additionally, a multinomial logistic regression model was performed to compare and contrast demographic and socio-economic predictors of cocaine-only and cocaine and opiate overdose with opiate-only overdose used as the referent category [32,33].

## Results

From 1990 through 2000, the OCME reported 8,774 fatal overdose deaths among New Yorkers aged 15–64 involving cocaine and/or opiates. Characteristics of total overdose, cocaine only overdose and opiate only overdose decedents in NYC are shown in Table 1. Among all overdose deaths during the study period, 2,392 (27.3%) were attributed to cocaine only and 2,825 (32.2%) were attributed to opiate only overdose.

**Table 1:** Bivariate associations of cocaine only, opiate only, and both cocaine and opiate overdose deaths among all accidental drug overdose decedents ages 15–64, New York City, 1990–2000*

	Total		Cocaine only overdose deaths			Opiate only overdose deaths			Opiate and cocaine overdose deaths		
Characteristics	N	%	N	%	p-value	N	%	p-value	N	%	p-value
Total	8774	100.0	2392	27.3	---	2825	32.2	---	3557	40.5	---
Sex											
Female	1807	20.6	630	34.9	<0.001	478	26.5	<0.001	699	38.7	0.071
Male	6967	79.4	1762	25.3		2347	33.7		2858	41.0	
Race/ethnicity											
White	2948	33.6	486	16.5	<0.001	1242	42.1	<0.001	1220	41.4	<0.001
Black	3234	36.9	1348	41.7		671	20.7		1215	37.6	
Hispanic	2592	29.5	558	21.5		912	35.2		1122	43.3	
Age											
15–24	419	4.8	70	16.7	<0.001	170	40.6	<0.001	179	42.7	<0.001
25–34	2369	27.0	573	24.2		704	29.7		1092	46.1	
35–44	3788	43.2	977	25.8		1242	32.8		1569	41.4	
45–54	1834	20.9	602	32.8		609	33.2		623	34.0	
55–64	364	4.1	170	46.7		100	27.5		94	25.8	
Borough of death											
Manhattan	2798	32.0	769	27.5	0.085	783	28.0	<0.001	1246	44.5	<0.001
Bronx	2110	24.1	579	27.4		641	30.4		890	42.2	
Brooklyn	2391	27.3	684	28.6		850	35.5		857	35.8	
Queens	1273	14.6	311	24.4		469	36.8		493	38.7	
Staten Island	176	2.0	49	27.8		82	46.6		71	40.3	
Cannabis detected											
no	8076	92.0	2248	27.8	<0.001	2602	32.2	0.880	3226	39.9	<0.001
yes	698	8.0	144	20.6		223	31.9		331	47.4	
Alcohol detected											
no	5070	57.8	1663	32.8	<0.001	1460	28.8	<0.001	1947	38.4	<0.001
yes	3704	42.2	729	0.2		1365	36.9		1610	43.5	
Place of OD episode											
Other inside	1122	13.4	257	22.9	<0.001	363	32.4	0.002	502	44.7	0.015
Residence	6359	75.7	1697	26.7		2109	33.2		2553	40.1	
Outside	918	10.9	291	31.7		250	27.2		377	41.1	
Day of the week											
Monday-Thursday	4647	53.0	1306	28.1	0.061	1484	31.9	0.586	1857	40.0	0.240
Friday-Sunday	4127	47.0	1086	26.3		1341	32.5		1700	41.2	
Year of death											
1990	506	5.8	148	29.2	<0.001	155	30.6	<0.001	203	40.1	<0.001
1991	708	8.1	247	34.9		184	26.0		277	39.1	
1992	772	8.8	233	30.2		210	27.2		329	42.6	
1993	1014	11.6	241	23.8		288	28.4		485	47.8	
1994	994	11.3	283	28.5		266	26.8		445	44.8	
1995	979	11.2	231	23.6		317	32.4		431	44.0	
1996	852	9.7	250	29.3		290	34.0		312	36.6	
1997	806	9.2	219	27.2		314	39.0		273	33.9	
1998	737	8.4	193	26.2		251	34.1		293	39.8	
1999	651	7.4	169	26.0		247	37.9		235	36.1	
2000	755	8.6	178	23.6		303	40.1		274	36.3	

*analysis restricted to decedents with opiates-only, cocaine only, and both opiates and cocaine detected

Of the 8,774 fatal overdoses involving cocaine and/or opiates that occurred in NYC from 1990 to 2000, 79.4% were male, 33.6% were White, 36.9% were Black, and 29.5% were Hispanic. Nearly 75% of the decedents were younger than 45 years old at the time of death. Among the five boroughs of NYC, Manhattan had the largest proportion of accidental overdose deaths with 32.0% occurring in that borough; 27.3% of the total overdose fatalities occurred in Brooklyn, 24.1% in the Bronx, and smaller proportions in Queens (14.6%) and Staten Island (2.0%). While cannabis was detected in only 8.0% of the decedents at the time of death, alcohol was found in almost half (42.2%) of fatal overdoses. Most accidental overdose deaths occurred in a residence (75.7%). Almost one half of all overdose deaths occurred during a weekend (47.0%).

**Figure 1:**
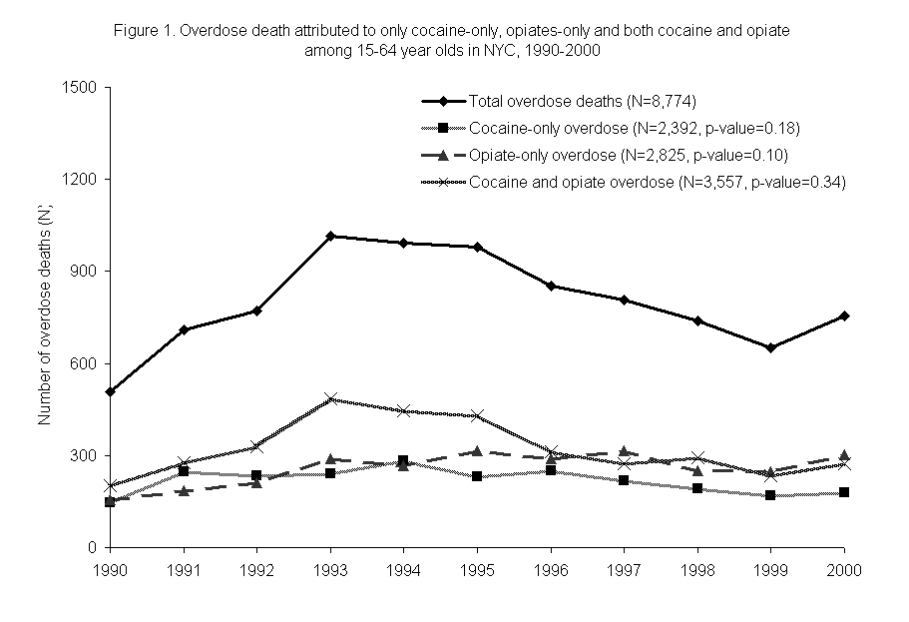
**Overdose death attributed to only cocaine-only and opiate only among 15–64 year olds in NYC, 1990–2000 (N = 8,774).** Cocaine only overdose deaths were decedents in which cocaine (not in the presence of opiates) was the cause of death; other drugs (except opiates) may also have been contributors of cause of death; Opiate only overdose deaths were decedents in which opiates (without the presence of cocaine) were the cause of death; other drugs (except cocaine) may also have been contributors of cause of death. Cocaine and opiate overdose deaths refers to overdose deaths in which both cocaine and opiates were the cause of death; other drugs may also have been contributors of cause of death.

The total number of accidental overdose deaths in NYC among 15–64 year olds related to cocaine and/or opiates increased from 1990 to 1993, plateaued, and then exhibited a steady decline until 1999 (Figure 1). A slight increase in the total fatal overdoses in this population was seen in 2000. While the number of cocaine only overdose deaths remained relatively constant from 1990–2000 (χ^2 ^test for trend, p = 0.18), opiate only deaths saw a small, but steady increase during the same time period (χ^2 ^test for trend, p = 0.10).

**Figure 2:**
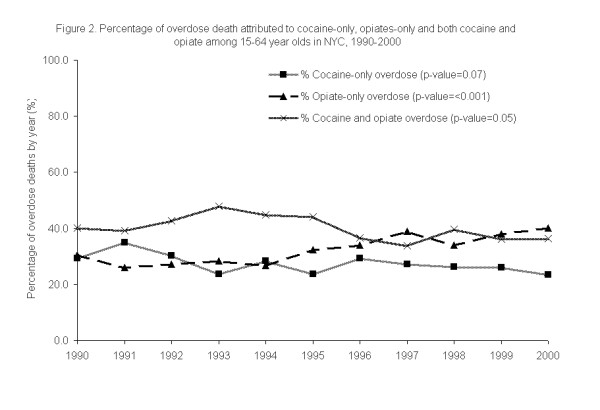
**Percentage of drug overdose death attributed to cocaine only and opiates-only among 15–64 year olds in NYC, 1990–2000 (N = 8,774).** Cocaine only overdose deaths were decedents in which cocaine (not in the presence of opiates) was the cause of death; other drugs (except opiates) may also have been contributors of cause of death; Opiate only overdose deaths were decedents in which opiates (without the presence of cocaine) were the cause of death; other drugs (except cocaine) may also have been contributors of cause of death. Cocaine and opiate overdose deaths refers to overdose deaths in which both cocaine and opiates were the cause of death; other drugs may also have been contributors of cause of death.

Figure 2 shows the trends in the proportion of total accidental overdose deaths that involved cocaine and/or opiates. While the absolute number of cocaine only fatal overdoses remains constant from 1990–2000, the proportion of overdoses attributed to cocaine-only declined during the 11-year period from 29.2% to 23.6% (χ^2 ^test for trend, p = 0.07). Over the same period, the proportion of opiate only fatal overdoses rose from 30.6% in 1990 to 40.1% in 2000 (χ^2 ^test for trend, p=<0.001).

The descriptive characteristics of the three groups of decedents are shown in Table 1. Among women overdose decedents, 34.9% were classified as cocaine-only compared to 25.3% among men with a fatal overdose. This trend was reversed for opiate-only and cocaine and opiate overdose deaths. Among Black overdose decedents 41.7% were cocaine only, 20.7% opiate only, and 37.6% cocaine and opiate fatal overdoses. For Whites who fatally overdosed, the trend is different, with the largest proportion categorized as opiate (42.1%); the largest proportion of Hispanic decedents was cocaine and opiate overdose deaths (43.3%). A dose response was seen with respect to age with likelihood of cocaine only overdose among all overdose deaths, increasing with age (p < 0.001). Borough of death was associated with all three categories, with cocaine only and opiate only overdose fatalities occurring in the largest proportion for decedents residing in all boroughs except Staten Island. The absence of either cannabis (27.8% versus 20.6% for cannabis detected) or alcohol (32.8% versus 0.2% for alcohol detected) was associated with an increased likelihood of a cocaine only overdose (p < 0.001) among those fatally overdosing in NYC. All three groups had associations with location of overdose.

The results of multivariate logistic regression models for the three categories of overdose decedents are shown in Table 2. The first model compared those with a cocaine only overdose death to those with a fatal overdose in the two other categories. Decedent males were less likely to experience a cocaine only overdose than females (adjusted OR = 0.77, 95% CI 0.68–0.87). Furthermore, both Black (adjusted OR = 3.23, 95% CI 2.85–3.67) and Hispanic (adjusted OR = 1.37, 95% CI 1.19–1.58) overdose fatalities were more likely to be from cocaine only, compared to Whites. Decedents at older ages were more likely to have fatally overdosed from cocaine only than from some other drug(s). No associations were found between borough of death, cannabis detection, or year of death and cocaine only overdose. However, a negative association was found between alcohol presence at autopsy and cocaine only fatal overdose.

**Table 2:** Multivariate logistic regression models of cocaine only and opiate only and both cocaine and opiate overdose deaths among all accidental drug decedents ages 15–64, New York City, 1990–2000*

	Cocaine only overdose deaths		Opiate only overdose deaths		Cocaine and opiate overdose deaths	
	Adjusted		Adjusted		Adjusted	
Characteristics	OR	95 CI	OR	95 CI	OR	95 CI
Sex						
Female	REF	---	REF	---	REF	---
Male	0.77	0.68–0.87	1.22	1.08–1.38	1.05	0.94–1.17
Race/ethnicity						
White	REF	---	REF	---	REF	---
Black	3.23	2.85–3.67	0.36	0.32–0.41	0.94	0.84–1.04
Hispanic	1.37	1.19–1.58	0.78	0.69–0.87	1.06	0.94–1.18
Age						
15–24	REF	---	REF	---	REF	---
25–34	1.41	1.07–1.88	0.68	0.54–0.84	1.15	0.93–1.43
35–44	1.35	1.02–1.78	0.86	0.69–1.06	0.98	0.80–1.21
45–54	1.81	1.36–2.41	0.92	0.73–1.15	0.73	0.58–0.91
55–64	2.95	2.09–4.17	0.80	0.58–1.09	0.49	0.36–0.67
Borough of death						
Manhattan	REF	---	REF	---	REF	---
Bronx	1.02	0.89–1.17	1.13	0.99–1.28	0.90	0.80–1.02
Brooklyn	1.04	0.92–1.19	1.46	1.29–1.65	0.70	0.63–0.79
Queens	1.05	0.89–1.24	1.34	1.15–1.55	0.75	0.66–0.86
Staten Island	1.28	0.90–1.81	1.30	0.96–1.76	0.67	0.49–0.90
Place of OD episode						
Other inside (not residence)	REF	---	REF	---		
Residence	0.96	0.84–1.10	1.09	0.96–1.23		
Outside	1.27	1.05–1.54	0.86	0.71–1.04		
Cannabis detected						
no	REF	---			REF	---
yes	0.85	0.69–1.03			1.27	1.08–1.49
Alcohol detected						
no	REF	---	REF	---	REF	---
yes	0.53	0.48–0.59	1.39	1.26–1.53	1.20	1.10–1.32
Year of death						
1990	REF	---	REF	---	REF	---
1991	1.25	0.96–1.62	0.80	0.62–1.04	0.99	0.78–1.25
1992	1.12	0.86–1.45	0.77	0.61–1.00	1.12	0.89–1.42
1993	0.80	0.62–1.03	0.81	0.64–1.03	1.39	1.12–1.73
1994	0.98	0.76–1.26	0.76	0.60–0.97	1.27	1.02–1.58
1995	0.74	0.58–0.96	1.00	0.79–1.27	1.23	0.99–1.53
1996	1.01	0.78–1.30	1.05	0.83–1.35	0.95	0.75–1.19
1997	0.90	0.69–1.17	1.34	1.05–1.71	0.82	0.65–1.04
1998	0.85	0.65–1.11	1.03	0.81–1.33	1.10	0.87–1.39
1999	0.85	0.65–1.12	1.21	0.94–1.57	0.94	0.73–1.20
2000	0.74	0.57–0.97	1.31	1.03–1.68	0.96	0.76–1.22

*analysis restricted to decedents with opiates-only, cocaine only, and both opiates and cocaine detected

The second model shown in Table 2 examined opiate only fatal overdoses compared to the other two groups. Here, males were more likely to experience an opiate only than any other drug combination (adjusted OR = 1.22, 95% CI 1.08–1.38). Contrary to what was found with respect to cocaine only overdose fatalities, for drug decedent New Yorkers, Whites were more likely to have an opiate only than Hispanics or Blacks. After adjustment for other covariates, the negative association with age all but disappeared. When borough of death was examined (borough of Manhattan used as the referent category), those New Yorkers fatally overdosing in Brooklyn (adjusted OR = 1.46, 95% CI 1.29–1.65) or Queens (adjusted OR = 1.34, 95% CI 1.15–1.55), decedents were more likely to have had an opiate only overdose. Alcohol detection at autopsy was associated with a nearly 40% increase in the likelihood that a New Yorker fatally overdosed on opiates-only, compared to other fatal drug combinations.

No gender or race associations were found when cocaine and opiate fatal drug overdoses were modeled. Decedents over 45 years old were less likely to have overdosed on a combination of cocaine and opiates. When compared to Manhattan, decedent in Brooklyn, Queens, or Staten Island were less likely to have had an overdose attributed to cocaine and opiates. Both cannabis and alcohol detection was associated with an increased likelihood of overdose death from cocaine and opiates, compared to overdose death attributed to other drug combinations.

Table 3 shows the results of the single multinomial regression model in which cocaine only is directly compared to opiate only overdose deaths. Male decedents were less likely to have overdosed on cocaine only than opiates-only. Additionally, Blacks who fatally overdosed were nearly five times more likely to have done so from cocaine only; Hispanics were 1.5 times more likely. Age at death greater than 24 was associated with an increased likelihood that the fatal overdose was attributed to cocaine only rather than opiates-only. Overdose death in Brooklyn was more likely a result of opiates-only than cocaine only. Fatal overdoses that occurred outside were more likely to have been attributed to cocaine only than opiates only. Alcohol detected at autopsy was half as likely among those who overdosed on cocaine only. There was no significant association observed for overdose deaths from cocaine only with respect to year of death.

**Table 3:** Multinomial logistic regression comparing cocaine only and both opiate and cocaine overdose compared to opiate only overdose deaths among decedents ages 15–64, New York City, 1990–2000*

	Cocaine only overdose deaths vs opiate only overdose deaths			Opiate/cocaine overdose deaths vs opiate only overdose deaths		
	Adjusted model			Adjusted model		
Characteristics	OR	95 CI	p-value	OR	95 CI	p-value
Sex						
Female	REF	---	---	REF	---	---
Male	0.71	0.62 – 0.82	<0.0001	0.88	0.77 – 1.01	0.0733
Race/ethnicity						
White	REF	---	---	REF	---	---
Black	4.73	4.08 – 5.49	<0.0001	1.94	1.71 – 2.21	<0.0001
Hispanic	1.51	1.29 – 1.76	<0.0001	1.20	1.06 – 1.36	0.0035
Age						
15–24	REF	---	---	REF	---	---
25–34	1.74	1.27 – 2.37	0.0005	1.41	1.12 – 1.79	0.0040
35–44	1.43	1.05 – 1.94	0.0217	1.11	0.88 – 1.39	0.3891
45–54	1.69	1.23 – 2.32	0.0012	0.88	0.69 – 1.13	0.3318
55–64	2.53	1.70 – 3.75	<0.0001	0.75	0.52 – 1.08	0.1254
Borough of death						
Manhattan	REF	---	---	REF	---	---
Bronx	0.93	0.79 – 1.10	0.3915	0.87	0.76 – 1.00	0.0551
Brooklyn	0.80	0.68 – 0.93	0.0033	0.63	0.55 – 0.72	<0.0001
Queens	0.85	0.71 – 1.03	0.0934	0.70	0.60 – 0.82	<0.0001
Staten Island	1.01	0.69 – 1.49	0.9575	0.66	0.47 – 0.93	0.0165
Place of OD episode						
Other inside (not residence)	REF	---	---	REF	---	---
Residence	0.92	0.78 – 1.07	0.2830	0.93	0.81 – 1.06	0.2754
Outside	1.34	1.06 – 1.68	0.0127	1.08	0.88 – 1.32	0.4626
Cannabis detected						
no	REF	---	---	REF	---	---
yes	0.96	0.77 – 1.21	0.7590	1.25	1.04 – 1.50	0.0161
Alcohol detected						
no	REF	---	---	REF	---	---
yes	0.50	0.44 – 0.56	<0.0001	0.89	0.80 – 0.98	0.0221
Year of death						
1990	REF	---	---	REF	---	---
1991	1.37	1.00 – 1.87	0.0465	1.17	0.88 – 1.55	0.2790
1992	1.30	0.95 – 1.77	0.0963	1.28	0.97 – 1.68	0.0831
1993	0.98	0.72 – 1.31	0.8726	1.38	1.06 – 1.78	0.0157
1994	1.19	0.89 – 1.60	0.2449	1.38	1.06 – 1.79	0.0161
1995	0.79	0.59 – 1.07	0.1281	1.11	0.86 – 1.44	0.4269
1996	0.97	0.72 – 1.30	0.8246	0.93	0.71 – 1.21	0.5770
1997	0.76	0.56 – 1.03	0.0727	0.73	0.56 – 0.95	0.0210
1998	0.86	0.63 – 1.18	0.3552	1.03	0.78 – 1.35	0.8522
1999	0.78	0.57 – 1.07	0.1178	0.84	0.64 – 1.11	0.2271
2000	0.66	0.49 – 0.91	0.0098	0.81	0.62 – 1.06	0.1276

*analysis restricted to decedents with opiates-only, cocaine only, and both opiates and cocaine detected

While no gender association was observed from the multinomial regression model with respect to cocaine and opiate fatal overdose (Table 3), both Blacks and Hispanics who fatally overdosed were more likely to have the fatality attributed to cocaine and opiates than opiates-only. There was a weak trend towards older decedents being less likely to overdose on cocaine and opiates. Death in the boroughs of Brooklyn, Queens or Staten Island was inversely associated with a cocaine and opiates fatal overdose. Detection of alcohol at autopsy was negatively associated with cocaine and opiates overdose, while cannabis detection at autopsy showed a positive association.

## Discussion

We examined all accidental drug overdose deaths involving cocaine and/or opiates that occurred in NYC between 1990 and 2000. While the absolute number of cocaine only overdose deaths remained relatively stable throughout the period, the number of opiate only fatal overdoses rose slightly. Of all overdose deaths during the study period, 27.3% were attributed to cocaine only and 32.2 to opiates-only. Decedents who had fatal cocaine only overdoses appear to differ from those who had opiate only overdoses. This suggests that those New Yorkers who die from accidental cocaine- or opiate only overdoses represent two distinct populations, requiring different interventions aimed at prevention.

While the number of cocaine only overdoses was stable over the decade examined, cocaine only overdoses accounted for a quarter of total fatal overdoses. This number is considerably higher when polydrug overdoses involving both cocaine and opiates are included, underscoring the contribution of cocaine abuse to overdose deaths in NYC. Accidental fatal overdose is possible with even a relatively low concentration of cocaine [34-36]. Furthermore, cocaine use is not restricted to injectors only. While less common than injecting users, non-injection cocaine users do experience overdoses [26]. Intranasal cocaine abuse among affluent, employed populations also has been associated with fatal overdoses [37-39]. Although our dataset does not allow us to ascertain different modes of cocaine administration, the contribution of cocaine to overall overdose deaths is unmistakable and has clear implications for overdose prevention efforts that predominantly target opiate use only [17,20,40,41].

There were several important differences between cocaine only and opiate only decedent populations. In the multiple logistic regression models, female decedents were more likely to have a cocaine only overdose and male decedents an opiate only overdose. This finding is contrary to other studies [25,42] and particularly interesting since it has been shown that males with a fatal cocaine overdose were more likely to have contributory coronary disease[25]. The higher risk of cocaine only overdose among women seen in our analysis may reflect unique characteristics of the drug economy and drug use patterns in the United States or in NYC and bears further study.

Our results suggest that cocaine only and opiate only overdose decedents represent two substantially different population groups. For cocaine only overdose fatalities, minorities (Blacks and Hispanics) were at a significantly higher risk. These findings have been confirmed in previous analyses[10]. There were also geographic differences in the locations of cocaine only vs. opiate only deaths in NYC. Among overdose fatalities, being found to have an opiate only overdose death was associated with residence in Brooklyn or Queens. When cocaine only and opiate only fatal overdoses were examined in the multinomial regression model, only death in Brooklyn showed a significant association. To some degree, these disparities may reflect differences in drug use patterns among different racial/ethnic groups in certain boroughs of the city. Consistent with our observations, other studies have shown that in NYC, opiate use occurs largely among younger Whites [43] and that nationally, cocaine use was more likely among minorities than among Whites[44].

The median age of both cocaine only and opiate only decedents was between 34–44. In the multinomial regression model, older age at fatal overdose was associated with a cocaine only fatal overdose. Furthermore, the data were suggestive of increasing likelihood of a cocaine only overdose with increasing age. While we do not have data on the age of initiation of cocaine use in this dataset, the finding of an increasing risk of overdose with increasing age may suggest either that newer users are less likely to experience a fatal overdose or that younger persons were more likely to use opiates than cocaine. It is also possible that pre-existing medical conditions, such as atherosclerosis and heart disease, which occur more commonly in older individuals[45], may be exacerbated by cocaine use, even in moderate doses.

The location of the overdose episode was not associated with an opiate only-induced fatality. However, an overdose outside (compared to a non-residential indoor location) was positively associated with a cocaine only accidental overdose death. The lack of an association between location of overdose episode and opiate only death may be explained by the fact that many opiate users have experienced or witnessed an overdose[20] and may be more apt to intervene with either life-saving measures or contact of emergency personnel. This finding is particularly relevant as more interventions targeting opiate overdose are implemented [46].

The presence of alcohol at autopsy was associated with an increased likelihood of an opiate only overdose, a finding that has been corroborated by others[12,13,22,47]. The trend was reversed however with respect to cocaine only overdose death, with the presence of alcohol being less likely. No association was found for cannabis presence for either cocaine only and opiate only accidental overdose fatalities. This finding underscores the poly-use nature of substance abuse. While we examined cocaine without the use of opiates and opiates without the use of cocaine for the purposes of understanding differences between these two drugs as contributors to overdose, we note that polydrug use was the predominant contributor of overdose deaths in this dataset.

In contrast to the findings of Darke et al [25] with respect to cocaine overdose death, we found no association with respect to overdose death occurring on the weekend versus a weekday for cocaine only or opiate only overdose death. This finding is consistent with the literature regarding heroin overdose[8,14]. It has been suggested that this finding may indicate a largely unemployed population, who use drugs on a regular basis as opposed to more recreational weekend abusers [25]. No consistent trend was identified with respect to year of death. The difference between our observations and those of Darke et al. may reflect geographic differences in patterns of cocaine use between the US and Australia, specifically the rare use of crack cocaine in Australia[48].

There are several limitations to this analysis. Determining the relative contribution of cocaine or opiates in the cause of death, when both drugs are ingested in concert is difficult since OCME determinations of cause of death are based on several factors, including (but not limited to) toxicology. The NYC OCME has standardized protocols for the classification of causes of death and all medical examiners were extensively trained. While likely small, variation among both medical examiners and among cases is possible, which may lead to misclassification of some of the data presented here. We present absolute numbers of overdose deaths and not rates in this analysis. Estimation of rates would require interpolation of denominator data. For example, to determine the rate of cocaine-only fatal overdoses would require an estimation of the total number of users of cocaine-only users in NYC. Data such as this is not readily accessible and likely would under-represent the number of true users. Thus, these data provide little information regarding the patterns of drug use, rather the patterns of fatal overdose. While we understand that drug use patterns vary by geography, age, and other factors, the objective of this analysis was to provide insight into the relative differences in overdose deaths from opiates and cocaine. Additionally, we have no information regarding the route of administration, dosage, or context of the opiates and cocaine. However, the fact that we do not have data regarding these factors should not obviate the findings we present here. Therefore, we present the absolute number of fatal overdose deaths, with the caveat that these data may not represent changes in the numbers of drug users in NYC.

This analysis excluded overdose deaths determined to be suicides by the OCME as well as fatal overdoses among those under the age of 15 and older than 65 and fatal overdoses not involving cocaine and/or opiates. Overdoses resulting from suicide likely represent a different set of behavioral mechanisms than accidental overdoses. Here we examine the differences between the characteristics of those accidental overdoses attributed to different drug combinations with the intent of informing interventions to reduce morbidity and mortality. Therefore, the addition of intentional (suicide) overdoses would have diminished our ability to examine correlates of accidental overdose. Furthermore, it is likely that the drug use patterns of individuals under 15 and over 65 are qualitatively different from those between 15 and 65, therefore we excluded them from analysis. The results of this study may not be generalizable outside of NYC, where using patterns may be dramatically different.

## Conclusion

This study is one of few that examine cocaine only fatal overdose and the only study we are aware of that describes the differences in decedents of opiate only and cocaine only overdose. Cocaine was an important contributor to accidental fatal overdose in NYC and was involved in nearly two-thirds of all decedents. Even though substantially more research has been published regarding opiate overdose than cocaine, we found that the proportion of overdose fatalities attributable to opiate only and cocaine only was similar throughout the decade. Additionally, these findings suggest that those who die from an opiate-only overdose are quite different from those who fatally overdose with cocaine only. The implications of these findings highlight the need to better understand the unique characteristics of different drug using populations, particularly in light of dynamic substance abuse epidemics[49]. Interventions designed to reduce overdose and its accompanying mortality that have proven successful in opiate-using populations may not work in populations of cocaine only users. Given the significant contribution of cocaine, both alone and in combination with other drugs to overdose mortality, a better understanding of the characteristics and risk factors for accidental fatality are critical for effective prevention.

## List of Abbreviations

NYC – New York City

OCME – Office of the Chief Medical Examiner

OR – Odds Ratio

CI – Confidence Interval

## Competing interests

The author(s) declare that they have no competing interests.

## Authors' contributions

KTB participated in the analysis and interpretation of the data and drafted the manuscript. AB and TMP participated in the analysis and interpretation of the data and assisted in revision of the manuscript. CG assisted in data acquisition and reviewed drafts of the manuscript. KT developed the study concept and design and reviewed drafts of the manuscript. SG developed the study concept and design, participated in data analysis and interpretation, reviewed drafts of the manuscripts and acted as senior author. All authors have read and approved the final version of this manuscript.

## Pre-publication history

The pre-publication history for this paper can be accessed here:


